# Laser Acupuncture and Heart Rate Variability—Scientific Considerations

**DOI:** 10.3390/medicines5020043

**Published:** 2018-05-07

**Authors:** Gerhard Litscher

**Affiliations:** Head of the TCM Research Center Graz, of the Research Unit of Biomedical Engineering in Anesthesia and Intensive Care Medicine, and of the Research Unit for Complementary and Integrative Laser Medicine, Medical University of Graz, 8036 Graz, Austria; gerhard.litscher@medunigraz.at; Tel.: +43-316-385-83907

This editorial analyzes current research trends and the effects of laser acupuncture on heart rate variability (HRV) and the autonomic nervous system, summarizing own research findings on the topic.

Laser acupuncture is a mostly non-invasive treatment method that has been scientifically evaluated in recent years, and whose treatment success has been documented in numerous high-level publications.

The present scientific research was carried out using the medical database PubMed (www.pubmed.gov). It searched for the terms “laser acupuncture and HRV”, and a total of *n* = 30 scientific papers were found. Exactly two-thirds (*n* = 20) of these research articles came from the author’s research group, and the latter included i.a. data from 233 patients, 42 healthy volunteers, and 49 experimental animals [1] (Figure 1).

The HRV is a neural control parameter of the heart that is widely used in scientific research and practice. This happens not only in Western, but also in evidence-based traditional medicine. Today, for the data acquisition and analysis of HRV in acupuncture, and especially in laser acupuncture, innovative recording technologies and artificial intelligence techniques are used for analysis [1].

Although all of the detailed mechanisms of HRV have not yet been fully elucidated, it is certain that intraindividual and interindividual variances exist, and that heart rate variation depends on age. In addition, circadian variations (sleep–wake cycle), physical condition, and mental and physical exertion are important influencing factors. HRV may also be affected by various conditions such as age and lifestyle-related diseases including diabetic neuropathy, renal failure, essential hypertension, heart disease, coronary heart disease, and intracranial lesions.

In China, electrocardiograms (ECGs) are registered with Austrian equipment, and the data is transferred to the Medical University of Graz via the internet immediately after laser acupuncture treatment. After the analysis of the ECGs is carried out in Graz, the acupuncturists in China are informed immediately of the results of the analysis, and the success of the therapy can be objectively demonstrated. Teleacupuncture forms a bridge function on the one hand between Eastern and Western medicine, and on the other between science and practice. In addition, there is a reduction in redundant research studies and a simplification of the diagnostic and therapeutic procedures. This not only saves costs, it also reduces time.

Our scientific research has clearly shown that special syndromes such as fatigue and stress can be counteracted with various preventative methods such as laser acupuncture. This has been shown in studies on patients with burnout syndrome in transcontinental teleacupuncture studies between China and Graz.

Teleacupuncture further demonstrates that under complex and critical circumstances, automated teletherapeutic procedures can be performed over a long distance. This could be useful under special circumstances (cooperation between experts from different continents), as our Sino-Austrian cooperation has shown.

A search in, for example, four Korean databases (RISS, NDSL, KISS and OASE with the terms “laser acupuncture” or “laser acupuncture point” or “laser therapy”, and “heart rate variability” or “HRV”, as well as Korean language terms for “laser acupuncture”) also provided 30 studies. These focused primarily on the effect of laser treatments on acupuncture points, the effect of transcutaneous laser irradiation, and reviews of the effectiveness of intravenous blood laser irradiation [2], including 14 studies with healthy volunteers and four studies with animal experiments that investigated the effects of laser acupuncture points. However, the points that were used for the studies were different; points that were often used included PC6, GV20, HT7, HT8, Liv3, LI4, LI11, BL21, and ST36.

Finally, it should be pointed out that certain devices for laser acupuncture have implemented different laser frequencies. Although this should indicate that at least someone has scientifically examined their validity, no one has done this so far. In a single scientific work, which can be found in the database PubMed, differences between the laser frequencies of 2 Hz and 100 Hz could be detected on an autonomic vegetative system [3,4].

In summary, due to the heterogeneity of the choice of acupuncture points, laser parameters, and study designs, the effect of the laser acupuncture on the neurovegetative system was different, and therefore, a conclusive assessment is not (yet) scientifically possible. Nevertheless, heart rate variability seems to be a very suitable method to quantify the vegetative effects of the laser acupuncture, and thus possibly control or document an evidence-based therapy.

## Figures and Tables

**Figure 1:**
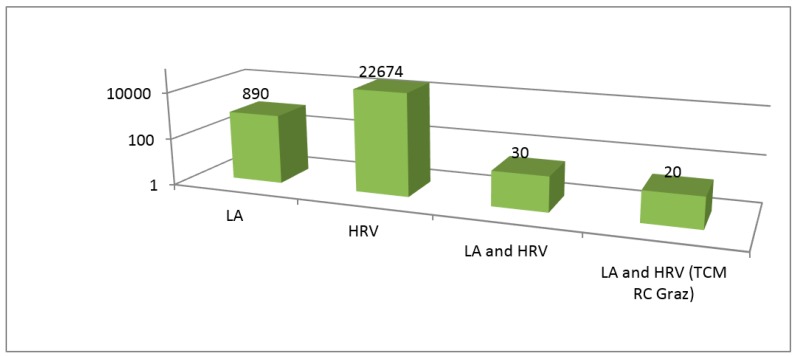
Number of scientific articles (PubMed, 3 May 2018) on laser acupuncture (LA) and heart rate variability (HRV). TCM RC Graz (Traditional Chinese Medicine Research Center Graz).

## References

[1] Litscher G. (2016). Heart Rate Variability and Acupuncture—Results from Transcontinental Studies.

[2] Lee S., Huh J.E. (2015). Research trends on the effect of laser acupuncture on heart rate variability. Integr. Med. Res..

[3] Robinson N.G. (2014). Laser acupuncture: Keep it scientific. Photomed. Laser Surg..

[4] He W., Wedig D., Wang L., Gaischek I., Litscher G. (2012). Violet laser acupuncture—part 5: An investigation of different stimulation frequencies on heart rate and variability. J. Acupunct. Meridian Stud..

